# Only the stable survive: role of balance task difficulty on dynamic postural control in young athletes and non-athletes

**DOI:** 10.3389/fspor.2025.1556727

**Published:** 2025-04-04

**Authors:** Thomas Muehlbauer, Katharina Borgmann, Sam Limpach, Dirk Krombholz, Adam Schweda, Sheila Geiger, Stefan Panzer

**Affiliations:** ^1^ Division of Movement and Training Sciences/Biomechanics of Sport, University of Duisburg-Essen, Essen, Germany; ^2^ Institute of Sport Science, Saarland University, Saarbrücken, Germany; ^3^ Dynamo Dresden, Dresden, Germany; ^4^ Clinic for Psychosomatic Medicine and Psychotherapy, LVR-University Hospital Essen, University of Duisburg-Essen, Essen, Germany; ^5^ Centre for Translational Neuro- and Behavioral Sciences (C-TNBS), University of Duisburg- Essen, Essen, Germany; ^6^ Department of Health and Kinesiology, Texas A&M University, College Station, TX, United States

**Keywords:** postural control, standing, task difficulty, sequential balance assessment, training experience, limb dominance

## Abstract

**Background:**

Emerging evidence highlights that adaptations in postural control induced by long-term motor practice are specific to the requirements in which the balance task is performed. In addition, the adaptations appear to be limb-specific, and as a result, may lead to differences in task failure when increasing the level of balance task difficulty. Thus, we determined differences in the percentage of participants with task failure (i.e., dropouts) during dynamic balance assessments for each limb while increasing the level of task difficulty in trained compared to untrained individuals.

**Methods:**

Soccer players (*n* = 64, age: 14.0 ± 1.8 years) with different levels of training experience (i.e., 2–5 or 6–9 years), swimmers (*n* = 73, age: 13.8 ± 2.7 years) and non-athletes (*n* = 60, age: 14.1 ± 1.1 years) performed the unipedal stance with the dominant and non-dominant leg under dynamic single (balance task only) and dual (balance and a concurrent motor task) task conditions with an increasing difficulty level (i.e., progressive reduction of the base of support). The percentage of participants remaining per completed difficulty level was analysed by type of sport, level of training experience, and leg dominance.

**Results:**

In both tasks, the percentage of individuals remaining per completed difficulty level decreased as the level of task difficulty increased, irrespective of the individual's training background. Further, significantly lower dropouts were found in soccer players compared to swimmers and non-athletes. However, no significant differences were detected between soccer players with diverging levels of training experience or with respect to limb dominance.

**Conclusions:**

The lower dropouts in soccer players compared to swimmers and non-athletes suggest sport-specific benefits in postural control for balance tasks with increasing difficulty level. However, this benefit is not superior in soccer players with more compared to less training experience and for the dominant than the non-dominant limb. These findings indicate that soccer players exhibit better postural control with both the dominant and non-dominant leg compared to swimmers and untrained individuals, which is maintained even with increasing balance task difficulty.

## 1 Introduction

Physical training leads to adaptations in the perception, transfer and processing of visual, vestibular and somatosensory information and consequently to enhanced balance performance (1). In this regard, Barone et al. (2) reported significantly lower centre of pressure (CoP) metrics (i.e., sway path, sway velocity) for the unipedal stance in soccer players compared to sedentary individuals. This finding is supported by a review article from Hrysomallis (3), who also reported better balance performance in athletes than in non-athletes. Additional evidence highlights that the above-mentioned adaptations are specific to the practiced type of sport. For example, Schwesig et al. (4) showed significantly lower sway values in the bipedal stance for shooting athletes compared to soccer players, handball players, and swimmers. Support for this finding is provided by works of Paillard (1, 5), stating sport-specific adaptations in postural control. Further research has also given considerable evidence for the existence of limb-specific adaptations in postural control. Ricotti et al. (6), for instance, investigated soccer players and found better balance performance for the standing compared to the kicking leg. This finding was attributed to the more frequent use of the standing leg to stabilize the body during soccer-related movements like passing, crossing, and kicking. Further empirical support is drawn largely from reviews (1, 7) reporting that postural control differs between the dominant and non-dominant leg, especially for athletes but less for untrained individuals.

Several assessment protocols have been used to test balance performance in conditions with varying levels of task difficulty. For example, in the sensory organization test, information is suppressed (e.g., closing the eyes to exclude vision) or manipulated (e.g., standing on foam ground to reduce proprioceptive precision) to increase the level of task difficulty (8). However, an increase in task difficulty can also be achieved by concurrently performing a secondary cognitive task (e.g., serial three subtractions) or reducing the base of support (e.g., unipedal stance) (9, 10). Yet, no studies have explored how differently trained vs. untrained individuals and trained individuals with varying levels of training experience control their posture in test conditions with progressively increased task difficulty and whether there are discrepancies between the dominant and non-dominant leg.

Given the information above, this study was designed to determine how postural control is affected by progressively increasing levels of balance task difficulty in trained and untrained individuals. Precisely, it was examined whether there are differences depending on the practiced type of sport, the level of training experience, and limb dominance. First, we hypothesised that with increasing level of balance task difficulty the percentage of individuals remaining per completed level will decrease, but the dropouts will be lower for athletes than for non-athletes, whereby athletes with (soccer) vs. without (swimming) balance requirements in their practiced sport will demonstrate the lowest dropouts. Second, we assumed that in soccer players, the percentage of players remaining per completed difficulty level depends on training experience, i.e., dropouts will be lower for players with more compared to less years of training. Third, we further expected that in soccer players there is also a dependency with regard to the examined leg, i.e., dropouts in players per difficulty level will be lower when testing the dominant than the non-dominant leg.

## 2 Material and methods

### 2.1 Participants

Sixty-four soccer players [20 females; age: 14.0 ± 1.8 years; height: 166.6 ± 11.3 cm; mass: 57.3 ± 12.5 kg, years from peak height velocity (PHV): −0.55 ± 1.33], 73 swimmers (40 females; age: 13.8 ± 2.7 years; height: 165.8 ± 13.9 cm; mass: 56.8 ± 14.6 kg, years from PHV: −0.32 ± 2.07), and 60 non-athletes (33 females; age: 14.1 ± 1.1 years; height: 165.2 ± 10.6 cm; mass: 61.5 ± 15.9 kg, years from PHV: −0.40 ± 1.04) participated in the present study after experimental procedures were explained. According to the findings and model for classifying the validity of expert samples in sport psychology research provided by Swann et al. (11), we differentiated between soccer players with less (2–5 years) or more (6–9 years) years of training experience at the athlete's highest level (i.e., national). All participants were free of any musculoskeletal dysfunction, neurological impairment, or orthopaedic pathology within the preceding three months. Participant's assent and written informed consent of the parents or legal guardians were obtained before the start of the study. Ethical approval (approval number: TM_04.06.2020) was obtained from the Human Ethics Committee at the University of Duisburg-Essen, Faculty of Educational Sciences.

### 2.2 Experimental procedure

All participants received standardised verbal instructions regarding the experimental procedure with a visual demonstration and familiarisation of all assessments. Afterwards, the following schedule was performed: (1) assessment of anthropometric variables; (2) execution of a standardised 10-min warm-up programme consisting of dynamic balance exercises; (3) assessment of dynamic balance performance in a random order. The same investigators (i.e., graduated sport scientists) supervised all assessments.

### 2.3 Assessments of anthropometric variables

Body mass was measured in light clothing and without shoes to the nearest 0.1 kg with an electronic scale (seca 803, Basel, Switzerland). The determination of body height was performed in an erect, outstretched posture, without shoes to the nearest 0.5 cm with a stadiometer (seca 217, Basel, Switzerland). The height represents the maximum distance between the top of the head and the ground.

### 2.4 Assessments of dynamic balance

**Figure 1:**
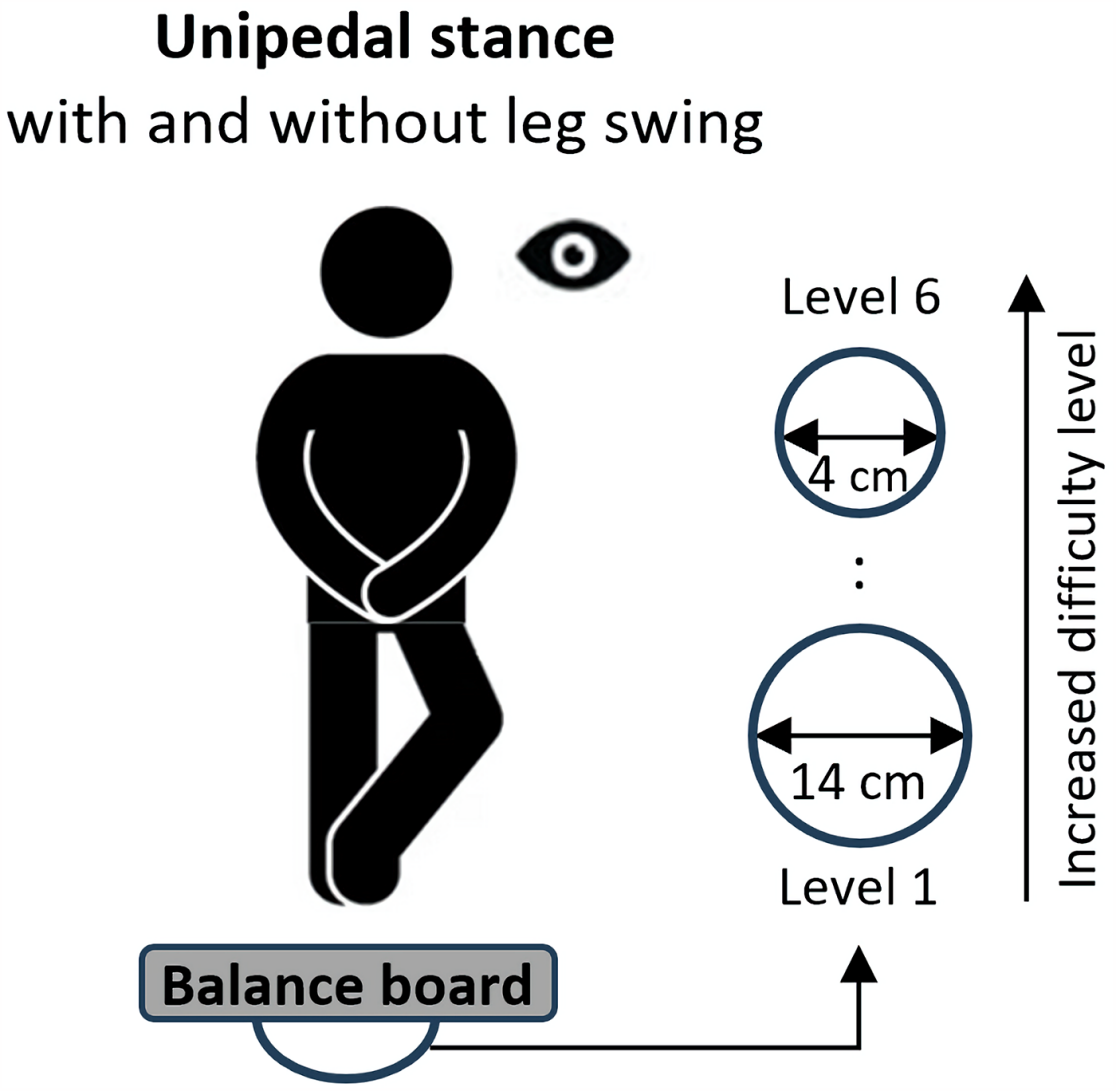
Schematic diagram for dynamic balance assessment. The balance board consists of an adjustable pivot to gradually reduce the base of support diameter from 14 cm (level 1) to 4 cm (level 6) Participants completed the balance tasks with progressively increased difficulty level.

Balance performance was assessed using the unipedal stance for the dominant and non-dominant leg (determined by self-report using the following question: “Which foot do you use to kick a ball?”) (7). Participants were asked to stand in erect position with hands placed on hips and gaze fixated on a cross over 30 s on a board with a mechanically adjustable pivot (Wobblesmart©, Artzt GmbH, Dornburg, Germany) that was placed on top of a force plate (Kistler; 9260AA, Winterthur, Switzerland) (12). For the *dynamic single task (i.e., balance task only)*, participants were instructed to stand as still as possible, whereas for the *dynamic dual task (i.e., balance and a concurrent motor task)* they were asked to move the contralateral leg back and forth by continuous hip flexion/extension and in accordance to a metronome (2 Hz) for six seconds (13) (Figure 1). The moving amplitude was set at 40 cm indicated by markers 20 cm in front and 20 cm behind the participant (13). While performing this task, the participants received auditory feedback from one experimenter who visually monitored their movement execution regarding movement amplitude and frequency to ensure that all participants execute the secondary motor task as accurately as possible. During both tasks, the difficulty level was increased by a progressive reduction of the base of support diameter [from level 1 (14 cm) = low difficulty to level 6 (4 cm) = high difficulty] of the mechanically adjustable pivot. The rationale for assessing dynamic balance performance with an increasing difficulty level was that postural control varies between individuals. It is therefore very likely that the results will be influenced by floor (too difficult) and ceiling (too easy) effects. Thus, the level of balance task difficulty was incrementally adjusted. Specifically, the adjustment was individualised for each participant to their maximum so that the participant could perform the last difficulty level either only with one leg and it was not possible to perform the same difficulty level with the other leg or – if the difficulty level was still manageable by both legs. Two practice and three data-collection trials were performed, and the mean was used for further analyses. A trial was discarded and repeated if participants (a) lose their balance (i.e., touch the ground with the non-stance leg), (b) remove the hands from the hips, (c) do not follow the metronome signal (during the dynamic balance task), or (d) do not reach the pre-set moving amplitude (dito). Depending on the task difficulty level, the reliability of the applied dynamic balance assessment ranged from ICC = .70–.97 for the single and from ICC = .64–.86 for the dual task condition, indicating “fair to good” to “excellent” values. Concurrent validity showed “excellent” values and ranged between ICC = .75 and .95, depending on the level of task difficulty. Furthermore, discriminant validity was detected for all levels (except for level 6) as indicated by significant differences between groups for the single task (all *p*-values ≤.003) and the dual task (all *p*-values ≤.038) condition.

### 2.5 Statistical analyses

Descriptive data were reported as group mean values *±* standard deviations (SD). To test the first hypothesis [i.e., role of sport (type)], we computed a set of generalized linear models with a binary outcome variable and a complementary log-log link function with the failure (i.e., failed to execute the timed unipedal stance test) at a specific difficulty level as a criterion, and the experience level (coded as a categorical variable), the group (soccer vs. swimming vs. non-athletes), their interaction term, and the participants' age and gender for both the dynamic single and dual task conditions. For both models, a participant-wise random intercept was added. Such model can be considered a discrete survival model because it captures the effect of the athletes' background on the difficulty-specific dropout. We refrained from considering more commonly used model types within the realm of survival analyses (particularly the Cox regression) due to the interval-censored nature of our dropout variable (i.e., some participants might have dropped out at a lower difficulty level while others dropped out at a higher level), as well as the comparatively low number of time points, which could lead to tied observations. We resorted to a full Bayesian framework due to convergence issues using standard methods from the R package lme4. Hence, we used the package brms (14) for computation, and the package emmeans (15) for illustration of the overall variable-wise effects (“joint tests”) and conditional effects. We, therefore, report *p*-values for the joint tests, but highest posterior density intervals (HPD) with a probability of 95% for the conditional effects comparisons. The only priors set were those for the regression coefficients, and these were comparably agnostic with a normal distribution with M = 0 and an SD of 1. For sampling, we used 24 chains with 2,000 iterations each.

To assess the second hypothesis (i.e., role of experience level), we computed models similar to the survival models and only included soccer players. Here, we created two groups based on the years of training experience (2–5 years vs. 6–9 years) and added the variable as a predictor into two further models including the failure during the single/dual dynamic balance task as a criterion. Hence, the difficulty level, the experience level, the interaction between these two as well as participants' age and gender, and a random intercept per participant served as predictors in the model.

To evaluate the third hypothesis (i.e., role of leg dominance), we computed two further models. As compared to the first set of models to assess the role of sport (type), where we collapsed the performance of both legs into one variable (i.e., a failure is coded already when the participant dropped out of the task with one leg), we used the leg-specific failure as a criterion and added the respective side (dominant leg vs. non-dominant leg) as a predictor, next to the difficulty variable (levels 1–6), the soccer players' age and gender. Again, separate models were computed for single and dual dynamic balance performance.

## 3 Results

### 3.1 Balance performance by difficulty level and type of sport

**Figure 2:**
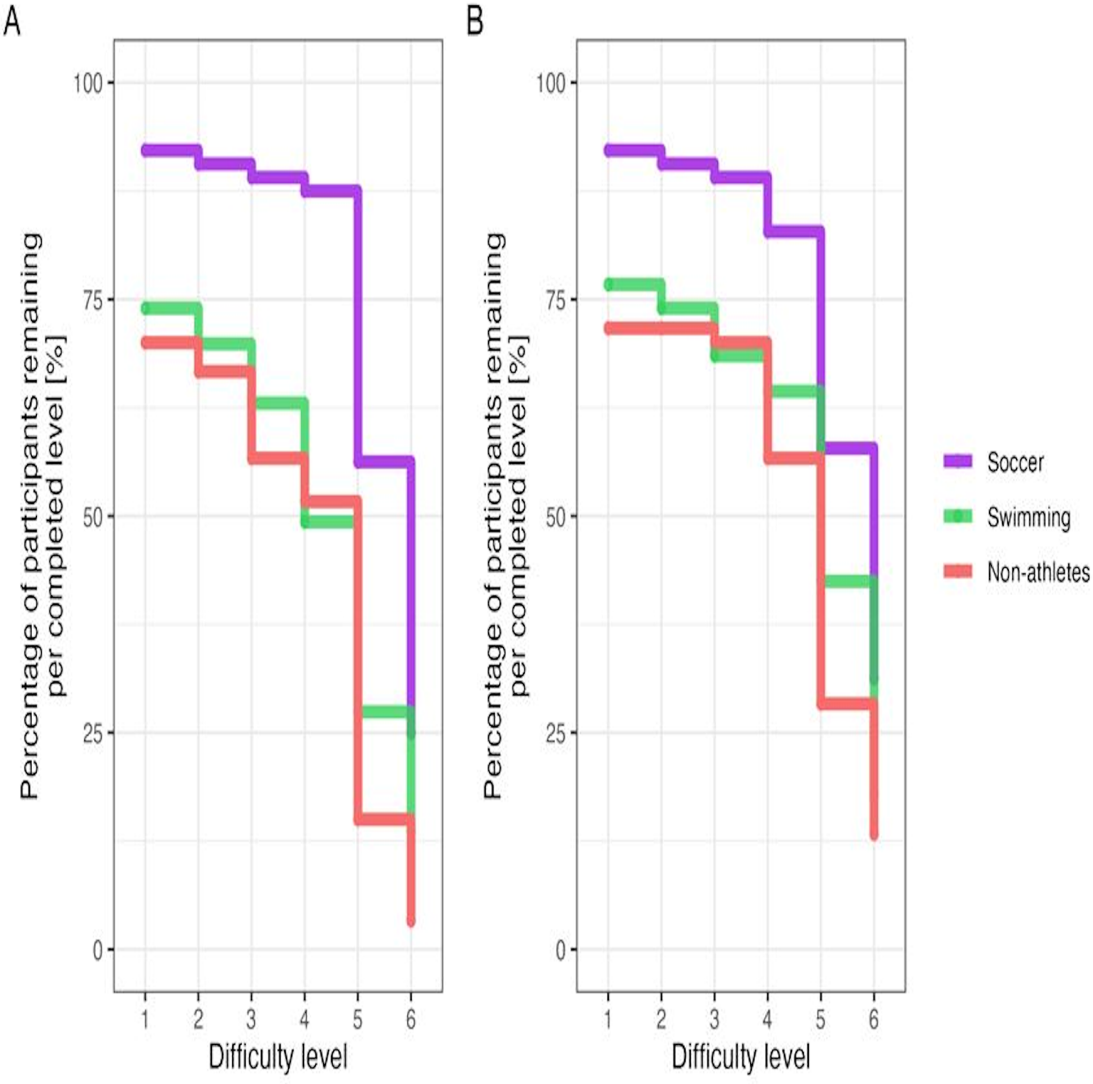
Percentage of participants remaining per completed difficulty level (i.e., 1–6) for the single **(A)** and dual **(B)** dynamic balance task by group (i.e., soccer, swimming, or non-athletes).

Figure 2 displays the percentage of participants remaining per completed difficulty level for the single (A) and dual (B) dynamic balance task by group. For the single dynamic balance task, the dropouts increased with increasing task difficulty level (Figure 2A). In this regard, we detected significant differences across difficulty levels [joint tests: χ^2^(5) = 177.560, *p* < .0001] and groups [joint tests: χ^2^ (2) = 20.914, *p* < 0.0001] as well as a significant interaction term [χ^2^ (10) = 20.590, *p* = 0.0242]. In terms of groups, tests for conditional effects showed a significantly lower dropout (i.e., critical difference) in soccer players compared to swimmers [estimate = 1.765, 95%-HPD interval = (0.733: 2.82)] and non-athletes [estimate = 2.359, 95%-HPD interval = (1.257: 3.45)]. Yet, no critical difference was detected between swimmers and non-athletes (estimate = 0.598, 95%-HPD interval = −0.597: 1.78). The significant interaction term might be generated by the increasingly diverging number of soccer players compared to non-athletes at higher difficulty levels. For instance, conditional effects analyses suggest estimated differences between soccer players and non-athletes at level 3 that amounted to 2.278 (95%-HPD interval = 0.898: 3.67), but reached 3.113 at level 6 (95%-HPD interval = 1.527: 4.78). Yet, due to the strong differences between groups, we believe that the main effects can be interpreted despite a significant interaction coefficient. No significant effects were found for age and gender.

For the dual dynamic balance task, we also found that the dropouts increased as the task difficulty level increased (Figure 2B). Again, we observed significant differences between difficulty levels [joint test: χ^2^ (5) = 154.59, *p* < .0001] and groups [χ^2^ (2) = 9.12, *p* = 0.011], yet no significant interaction term [χ^2^ (10) = 12.81, *p* = 0.234]. As for the single dynamic balance task, tests for conditional effects showed a significantly lower dropout (i.e., critical difference) in soccer players than in swimmers (estimate = 1.21, 95%-HPD interval = 0.0369: 2.40) and non-athletes (estimate = 1.75, 95%-HPD interval = 0.538: 2.97). Again, there was no critical difference between swimmers and non-athletes (estimate = 0.54, 95%-HPD interval = −0.877: 1.86).

### 3.2 Balance performance by difficulty level and level of training experience

**Figure 3:**
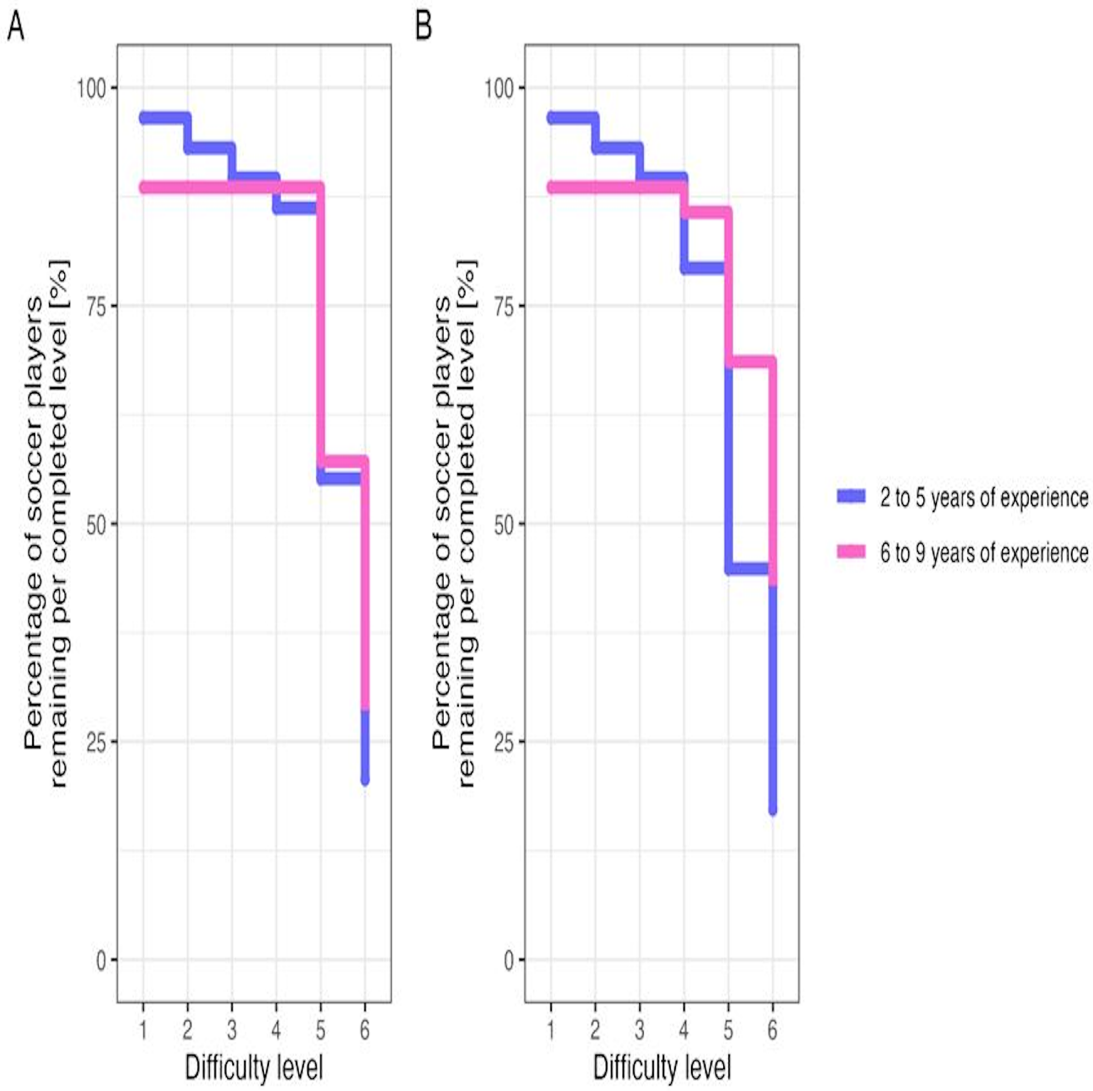
Percentage of soccer players remaining per completed difficulty level (i.e., 1–6) for the single **(A)** and dual **(B)** dynamic balance task by experience level (i.e., 2–5 years or 6–9 years).

Figure 3 shows the percentage of soccer players remaining per completed difficulty level for the single (A) and dual (B) dynamic balance task by experience level. For both balance tasks, the dropouts increased with increasing task difficulty level. We detected significant differences across difficulty levels [single, joint test: χ^2^(5) = 68.530, *p* < .0001; dual, joint test: χ^2^(5) = 66.460, *p* < .0001], but not between experience levels [single, joint test: χ^2^(1) = 0, *p* = 0.9884; dual, joint test: χ^2^(1) = 0.651, *p* = 0.4199]. Further, the interaction terms in both models did not reach the level of significance [single: χ^2^(5) = 4.135, *p* = .5302; dual: χ^2^(5) = 8.740, *p* = .1199].

### 3.3 Balance performance by difficulty level and limb dominance

**Figure 4:**
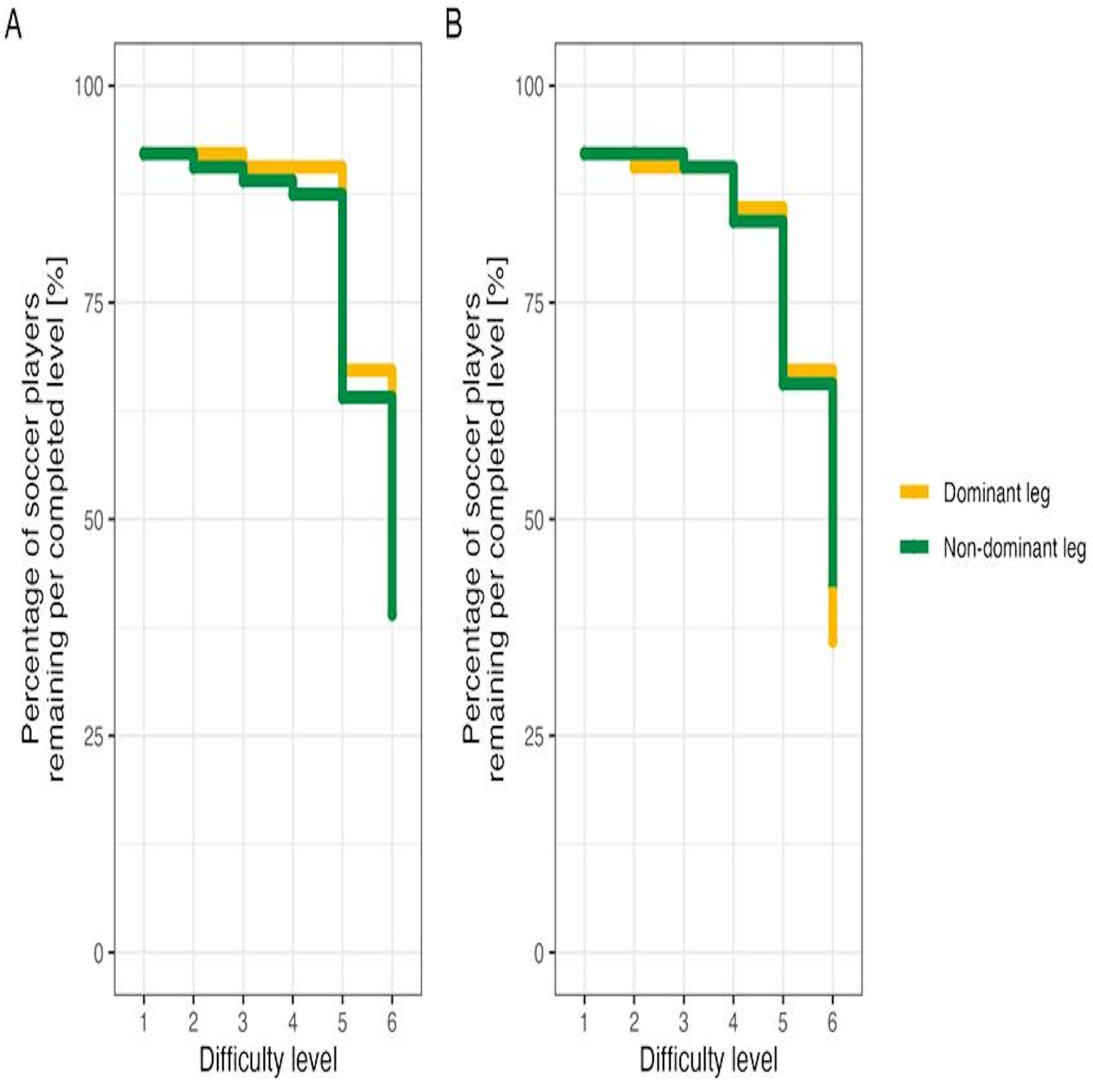
Percentage of soccer players remaining per completed difficulty level (i.e., 1–6) for the single **(A)** and dual **(B)** dynamic balance task by leg dominance (i.e., dominant or non-dominant).

Figure 4 illustrates the percentage of soccer players remaining per completed difficulty level (i.e., 1–6) for the single (A) and dual (B) dynamic balance task by leg dominance (i.e., dominant or non-dominant). Again, we found significant differences across difficulty levels [single, joint test: χ^2^(5) = 110.810, *p* < .0001; dual, joint test: χ^2^(5) = 104.475, *p* < .0001], but not with respect to limb dominance [single, joint test: χ^2^(1) = 0.718, *p* = .3969; dual, joint test: χ^2^(1) = 0.737, *p* = .3905]. In addition, the interaction terms in both models did not reached significance [single, joint test: χ^2^(5) = 1.765, *p* = .8808; dual, joint test: χ^2^(5) = 3.060, *p* = .6904].

## 4 Discussion

The present study was aimed to investigate how postural control is affected by progressively increasing the level of balance task difficulty in trained and untrained individuals. Three new findings emerged from this work: (1) in accordance with our first hypothesis, our results showed that soccer players showed less dropouts than swimmers and non-athletes when balance task difficulty was progressively increased, suggesting sport-specific benefits in postural control; (2) contrary with our second hypothesis, dropouts did not significantly differ between soccer players with diverging levels of training experience but the same competition level (i.e., national), indicating no experience-specific benefits in postural control; and (3) against our third hypothesis, dropouts were not significantly different in soccer players with respect to limb dominance and indicates no limb-specific benefits in postural control.

### 4.1 Balance performance and task difficulty: role of practiced type of sport

Consistent with our first hypothesis and previous research (10, 16, 17), increasing the level of balance task difficulty led to detrimental effects on postural control. With regard to the present study, we detected a decrease in the percentage of individuals remaining per completed difficulty level and dropouts were larger for untrained than trained individuals but were lowest in athletes with balance requirements in their practiced type of sport (soccer players). First, these findings are indicative of benefits in postural control when balance task difficulty is progressively increased for trained compared to untrained individuals. This is in line with a study by Biec and Kuczynski (18) who showed significantly better CoP metrics (i.e., variability, range, and velocity) for male soccer players compared to age-matched non-athletes during bipedal standing with eyes open (low difficulty level) and eyes closed (high difficulty level). The result of maintaining postural control despite increasing task difficulty could be attributed to several factors, including better functioning of sensors (i.e., visual, vestibular, proprioceptive and cutaneous receptors) relevant for balance (19–21), better central nervous processing (22–24), and better anticipatory (feedforward) and compensatory (feedback) postural control mechanisms (1).

In addition, the results provide evidence for sport-specific adaptations, because there were also significant differences between the trained individuals, i.e., lower dropouts (better postural control) in soccer players than in swimmers. This finding is supported by previous research (25) showing better balance performance in gymnasts compared to soccer players and swimmers when standing on an unstable platform. A possible reason could be that in soccer players compared to swimmers the unipedal stance (while passing, crossing, and kicking) is a situation that occurs repeatedly during training and competition and leads to sport-specific benefits in postural control (1). From a practical perspective, balance training is a suitable approach to improve postural control in young athletes and non-athletes (26).

### 4.2 Balance performance and task difficulty: role of training experience

In contrast to our second assumption, the number of dropouts did not significantly differ between soccer players with more (6–9 years) compared to less (2–5 years) years of training but the same competition level (i.e., national). This finding implies no expertise-specific benefits in postural control due to a higher level of training experience and is contrary to previous research (6, 27, 28) showing better balance performance in more compared to less experienced players. For example, Paillard et al. (28) assessed the unipedal stance in soccer players at different level of competition and found better postural performance in national compared to regional players. One reason to explain the discrepancy between our results and those of Paillard and colleagues might be that in the present study the time gap of 2–5 years vs. 6–9 years of training experience was not sufficient to induce structural and functional adaptations in the postural control system (1). In contrast, training experience in the study by Paillard et al. (28) was considerably higher and amounted to 10 ± 3 and 13 ± 2 years in regional and national players, respectively. Usually, more compared to less experienced athletes are physically stronger, show less movement variability and faster reaction times (6), which can have a positive effect on postural control. Since in the present study, the quantitative discrepancy in years of training did not show a significant difference in the number of dropouts when increasing the level of task difficulty during balance assessment, this might be generated by differences in the quality of training experience. Therefore, future work is needed to replicate our study and assess soccer players with the same duration but different levels (1st vs. 2nd vs. 3rd league players) of training experience. One might argue that the lack of training experience differences suggests the development of an early balance skill plateau and future research is necessary to reveal whether this is a general finding or specific to the tasks applied in the present study.

### 4.3 Balance performance and task difficulty: role of limb dominance

Contrary with our third expectation, the percentage of dropouts was not significantly affected by limb dominance in soccer players. This result contradicts the findings of Ricotti et al. (6) and Breen et al. (29), but is consistent with the those of Muehlbauer et al. (30) and Leinen et al. (13). The lack of significant differences between the limbs can have several causes. On the one hand, the selected test conditions may not have been specific enough to reflect the balance requirements in soccer. Although the unipedal stand and the continuous hip flexion/extension movements are tasks that represent situations such as crossing, passing and shooting, they are often performed under time pressure, with the influence of opponents and on turf. Therefore, future studies should examine whether an even stronger emphasis of the test requirements on the natural environmental conditions favours the detection of side differences. On the other hand, the level of expertise and the associated training experience may not have been sufficient to develop limb-specific structural and functional adaptations in the postural control system (1, 7). Precisely, Ricotti and colleagues (6) examined professional soccer players from the highest Italian soccer leagues (i.e., Serie A, Serie B etc.), while we analysed young sub-elite players from U13 to U17. In addition, unipedal actions such as crossing, passing and kicking only represent a comparatively small part of soccer training and game. In contrast, bipedal actions such as running, sprinting and jumping represent a large proportion of training and games in soccer (31) and may have counteracted the development of limb dominance.

The present study has some limitations that should be discussed. We conducted a cross-sectional study. Thus, no inferences about causal relationships can be drawn. Further, the unipedal stance was applied under single and dual dynamic balance task conditions and therefore our findings cannot be generalized to proactive (feedforward control) or reactive (feedback control) balance conditions or other tests. Moreover, task difficulty represents only one contextual factor. The investigation of additional factors such as task specificity by means of ecological (i.e., specific postural conditions related to the practiced type of sport) and non-ecological (i.e., decontextualised postural control conditions in relation to the practiced type of sport) test conditions would be quite valuable for future work. Lastly, balance performance was exclusively assessed on a behavioural but not on a neuromuscular level. Therefore, future studies should, for example, investigate muscle activation during balance assessment in order to expand our findings.

## 5 Conclusions

This study demonstrated that increasing the level of balance task difficulty during performing the unipedal stance under single and dual dynamic test conditions led to detrimental effects on postural control and that was irrespective of the individual's training background. The effects were less pronounced in soccer players compared to swimmers and non-athletes indicating the development of sport-specific benefits in postural control for balance tasks with increasing difficulty level induced by prolonged and repeated execution of soccer-specific movements such as passing, crossing, and kicking. It is therefore recommended to assess dynamic balance performance under sport-specific conditions and with progressively increasing levels of task difficulty. However, the effects on balance performance did not differ depending on the duration of training experience in soccer but could be influenced by its quality and thus be investigated in future studies. Further, limb dominance did not affect balance performance in soccer players, indicating no limb-specific benefits in postural control.

## Data Availability

The raw data supporting the conclusions of this article will be made available by the authors, without undue reservation.

## References

[1] Paillard T. Plasticity of the postural function to sport and/or motor experience. Neurosci Biobehav Rev. (2017) 72:129–52. 10.1016/j.neubiorev.2016.11.015 27894829

[2] Barone R Macaluso F Traina M Leonardi V Farina F Di Felice V. Soccer players have a better standing balance in nondominant one-legged stance. J Sport Med. (2011) 2:1–6. 10.2147/OAJSM.S12593 PMC3781875 24198563

[3] Hrysomallis C. Balance ability and athletic performance. Sports Med. (2011) 41(3):221–32. 10.2165/11538560-000000000-00000 21395364

[4] Schwesig R Kluttig A Leuchte S Becker S Schmidt H Esperer HD. The impact of different sports on posture regulation. Sportverletz Sportschaden. (2009) 23(3):148–54. 10.1055/s-0028-1109576 19750443

[5] Paillard T. Relationship between sport expertise and postural skills. Front Psychol. (2019) 10:1428. 10.3389/fpsyg.2019.01428 31293483 PMC6603331

[6] Ricotti L Rigosa J Niosi A Menciassi A. Analysis of balance, rapidity, force and reaction times of soccer players at different levels of competition. PLoS One. (2013) 8(10):e77264. 10.1371/journal.pone.0077264 24130870 PMC3795057

[7] Paillard T Noe F. Does monopedal postural balance differ between the dominant leg and the non-dominant leg? A review. Hum Mov Sci. (2020) 74:102686. 10.1016/j.humov.2020.102686 33059226

[8] McCollum G Shupert CL Nashner LM. Organizing sensory information for postural control in altered sensory environments. J Theor Biol. (1996) 180(3):257–70. 10.1006/jtbi.1996.0101 8759531

[9] Granacher U Bridenbaugh SA Muehlbauer T Wehrle A Kressig RW. Age-related effects on postural control under multi-task conditions. Gerontology. (2011) 57(3):247–55. 10.1159/000322196 20980734

[10] Muehlbauer T Roth R Bopp M Granacher U. An exercise sequence for progression in balance training. J Strength Cond Res. (2012) 26(2):568–74. 10.1519/JSC.0b013e318225f3c4 22067238

[11] Swann C Moran A Piggott D. Defining elite athletes: issues in the study of expert performance in sport psychology. Psychol Sport Exerc. (2015) 16:3–14. 10.1016/j.psychsport.2014.07.004

[12] Muehlbauer T Abel L Schedler S Panzer S. Acute effects of a single unilateral balance training session on ipsi- and contralateral balance performance in healthy young adults. BMC Res Notes. (2021) 14(1):356. 10.1186/s13104-021-05774-7 34507606 PMC8434721

[13] Leinen P Muehlbauer T Panzer S. Single-leg balance performance in sub-elite young soccer players and swimmers as a function of age and sports experience. J Motor Learn Dev. (2019) 7(3):374–88. 10.1123/jmld.2018-0055

[14] Bürkner PC. Brms: an R package for Bayesian multilevel models using stan. J Stat Softw. (2017) 80(1):1–28. 10.18637/jss.v080.i01

[15] Lenth R. emmeans: Estimated Marginal Means, aka Least-Squares Means. R package version 1.10.5. (2024). Available at: https://rvlenth.github.io/emmeans/, https://rvlenth.github.io/emmeans/ (Accessed January 20, 2025).

[16] Donath L Kurz E Roth R Zahner L Faude O. Leg and trunk muscle coordination and postural sway during increasingly difficult standing balance tasks in young and older adults. Maturitas. (2016) 91:60–8. 10.1016/j.maturitas.2016.05.010 27451322

[17] Barbado Murillo D Sabido Solana R Vera-Garcia FJ Gusi Fuertes N Moreno FJ. Effect of increasing difficulty in standing balance tasks with visual feedback on postural sway and EMG: complexity and performance. Hum Mov Sci. (2012) 31(5):1224–37. 10.1016/j.humov.2012.01.002 22658508

[18] Biec E Kuczynski M. Postural control in 13-year-old soccer players. Eur J Appl Physiol. (2010) 110(4):703–8. 10.1007/s00421-010-1551-2 20582432 PMC2957582

[19] Paillard T Bizid R Dupui P. Do sensorial manipulations affect subjects differently depending on their postural abilities? Br J Sports Med. (2007) 41(7):435–8. 10.1136/bjsm.2006.032904 17311808 PMC2465353

[20] Paillard T Noe F. Effect of expertise and visual contribution on postural control in soccer. Scand J Med Sci Sports. (2006) 16(5):345–8. 10.1111/j.1600-0838.2005.00502.x 16978254

[21] Vuillerme N Teasdale N Nougier V. The effect of expertise in gymnastics on proprioceptive sensory integration in human subjects. Neurosci Lett. (2001) 311(2):73–6. 10.1016/S0304-3940(01)02147-4 11567781

[22] Nielsen JB Cohen LG. The Olympic brain. Does corticospinal plasticity play a role in acquisition of skills required for high-performance sports? J Physiol. (2008) 586(1):65–70. 10.1113/jphysiol.2007.142661 17717010 PMC2375560

[23] Nakata H Yoshie M Miura A Kudo K. Characteristics of the athletes’ brain: evidence from neurophysiology and neuroimaging. Brain Res Rev. (2010) 62(2):197–211. 10.1016/j.brainresrev.2009.11.006 19944119

[24] Yarrow K Brown P Krakauer JW. Inside the brain of an elite athlete: the neural processes that support high achievement in sports. Nat Rev Neurosci. (2009) 10(8):585–96. 10.1038/nrn2672 19571792

[25] Davlin CD. Dynamic balance in high level athletes. Percept Mot Skills. (2004) 98(3 Pt 2):1171–6. 10.2466/pms.98.3c.1171-1176 15291203

[26] Gebel A Prieske O Behm DG Granacher U. Effects of balance training on physical fitness in youth and young athletes: a narrative review. Strength Cond J. (2020) 42(6):35–44. 10.1519/SSC.0000000000000548

[27] Butler RJ Southers C Gorman PP Kiesel KB Plisky PJ. Differences in soccer players’ dynamic balance across levels of competition. J Athl Train. (2012) 47(6):616–20. 10.4085/1062-6050-47.5.14 23182008 PMC3499884

[28] Paillard T Noe F Riviere T Marion V Montoya R Dupui P. Postural performance and strategy in the unipedal stance of soccer players at different levels of competition. J Athl Train. (2006) 41(2):172–6. 16791302 PMC1472651

[29] Breen EO Howell DR Stracciolini A Dawkins C Meehan WP. Examination of age-related differences on clinical tests of postural stability. Sports Health. (2016) 8(3):244–9. 10.1177/1941738116633437 26911999 PMC4981067

[30] Muehlbauer T Schwiertz G Brueckner D Kiss R Panzer S. Limb differences in unipedal balance performance in young male soccer players with different ages. Sports (Basel). (2019) 7(1):1–9. 10.3390/sports7010020 PMC6359298 30641997

[31] Morgans R Orme P Anderson L Drust B. Principles and practices of training for soccer. J Sport Health Sci. (2014) 3(4):251–7. 10.1016/j.jshs.2014.07.002

